# European innovation partnership on active and healthy ageing: triggers of setting the headline target of 2 additional healthy life years at birth at EU average by 2020

**DOI:** 10.1186/0778-7367-70-23

**Published:** 2012-10-22

**Authors:** Karolina Lagiewka

**Affiliations:** 1 Unit 02 - Innovation for Health and Consumers, Directorate General for Health and Consumer Policy, European Commission, Rue Belliard 232 7/41, Brussels, 1049, Belgium

**Keywords:** Healthy life years, Life expectancy, EU target, Compression, Expansion, Equilibrium, Disability, Morbidity, Mortality, Healthy ageing

## Abstract

**Background:**

The objective of this paper is to provide analytical research that supported the European Commission in setting the global target of additional two healthy life years (HLY) at birth by 2020 in the EU on average, within the European Innovation Partnership on Active and Healthy Ageing (the EIP on AHA). It produces a straightforward analysis of HLY projections that helped the European Commission set a firm, politically sound, target. In order to reach that goal, policy makers need to commit to redefining health priorities and goals and developing and implementing relevant strategies and programmes.

**Methods:**

The study computes a simple simulation of the HLY at birth based on three demographic scenarios: compression of morbidity, expansion of morbidity and an intermediary scenario, the dynamic equilibrium, given the expected 2.1 year gain in male and 1.6 in female life expectancy (LE) by 2020. Data on HLY and projections of life expectancy were obtained from Eurostat and 2008 was taken as a baseline. For consistency and given data gaps, EU27 average values of HLY were calculated.

**Results:**

In the EU27 as a whole, the difference between LE and HLY in 2008 was nearly 15 years for men and 20 years for women. The developments of healthy life expectancies across the EU Member States (MSs) are even more diverse that makes it difficult to model any robust EU level trends.

Under *compression of morbidity*, life expectancy and HLY would increase by 2020 on average by 2.1 and 2.0 years for men and by 1.6 and 1.4 years for women respectively. The expected years with disability would remain unchanged while the HLY/LE ratio would improve leading to a 0.5% gain for both genders. Under *expansion of morbidity*, life expectancy would increase by 2.1 years for men and 1.4 years for women by 2020, while HLY would remain unchanged and the expected years with disability would increase by 2.1 years and 1.6 years in women. This would imply the deterioration of the HLY/LE ratio for both men and women generating a 2.2% and 1.4% loss of health for men and women accordingly. Under *dynamic equilibrium*, the HLY would increase but to a lesser extent as the rise in life expectancy. The HLY would increase by 1.6 and 1.2 years for men and women respectively. HLY/LE ratio would remain unchanged for both men (+0.1%) and women. The study shows that the first scenario would reduce the HLY gap between the EU MSs by 1.4 years in men and 1.2 years in women, the second would generate no change, while the third one would reduce the gap by 0.9 years in men and increase it by 0.7 years in women.

**Conclusions:**

The results of the study triggered the political decision of setting the global target of 2 additional HLY for the European Innovation Partnership on Active and Healthy Ageing to be achieved by 2020. It is a ‘grand’ goal but can be achieved. Statistics clearly show that EU countries characterise very different levels of health progress, with a gap of 2 decades and diverging trends. With this in mind, the EU HLY target should be complemented by national HLY targets for men and women, set by MSs.

## Background

**Figure 1:**
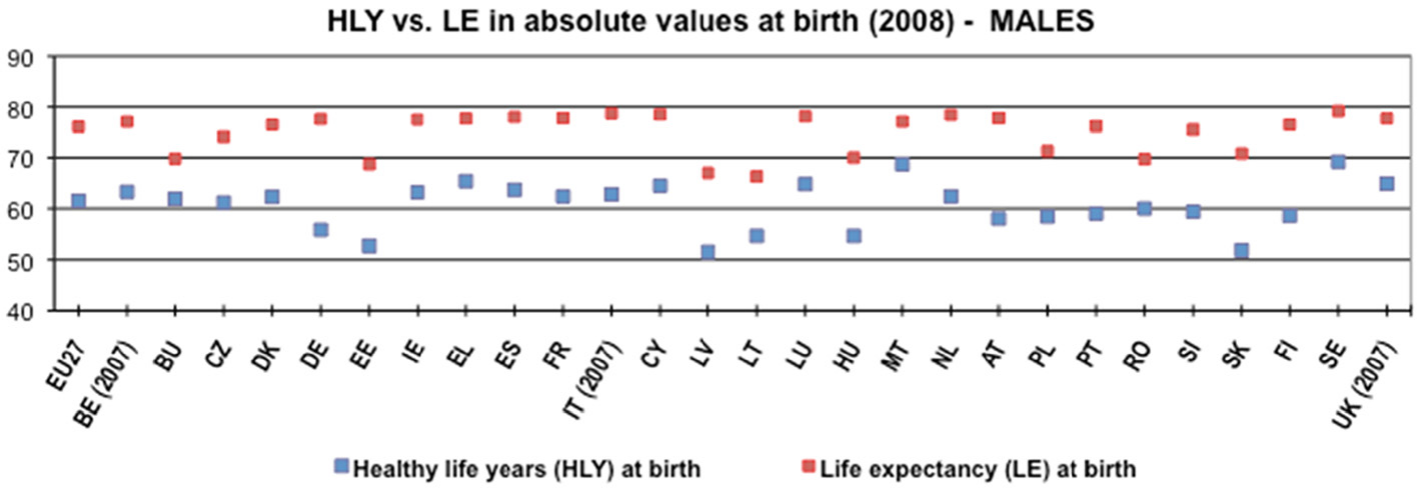
Life expectancy and healthy life years at birth among males within the European Union and Member States, 2008.

**Figure 2:**
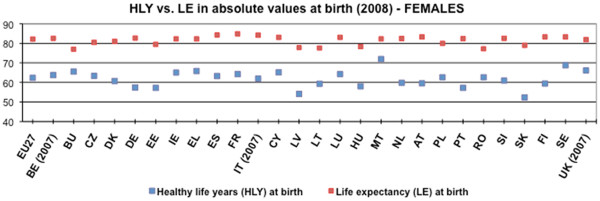
Life expectancy and healthy life years at birth among females within the European Union and Member States, 2008.

Europe and many other countries in the world are currently facing increasingly complex and systemic societal challenges. Due to health care advances, increased wealth, improved wellbeing and living standards and better diets life expectancy has increased dramatically [1]. It is projected that between 2010 and 2060 the number of Europeans aged over 65 will double, from 88 to 153 million, whilst of those over 80 will nearly triple, from 24 to 62 million [2]. However, the increased longevity has not always occurred in parallel with improved health and quality of life [3]. As demonstrated in Figures 1 and 2 there has been a considerable gap between the extended lifespan and the health expectancies. The ageing of the population has dominated demographic change as one of the most pertinent challenges of present and future.

In the light of the 1997 WHO Health Report, the Director-General of WHO, Dr. Hiroshi Nakajima stated that *increased longevity without quality of life is an empty prize. Health expectancy is more important than life expectancy*. The experience of the European Union (EU) underlines the need to focus on health. Health and healthy population is fundamental to the pursuit of smart, sustainable and inclusive growth and better jobs. More healthy life years mean a healthier workforce, and less retirement on the grounds of ill health. It reduces the burden on formal and informal care structures, leading to less strain on public finances and contributing to the longer-term sustainability of the health and social systems as the population ages [4].

A range of factors impact the health status of ageing populations therefore it cannot be simply assumed how the healthy life expectancy (disability trends) will develop in next decades. For example, rising obesity might cause future increases in unhealthy lifespan, whereas improvements in medical technologies such as joint replacements can contribute towards lower disability rates and higher healthy life years [2]. Assumptions, therefore, cannot be made on the development of morbidity and disability in the next decades, and on the interaction between declining mortality, morbidity and disability. Such uncertainty over health and disability trends, combined with current data limitations, entails the need to model different scenarios. For almost 50 years, there has been much debate over whether people will live longer, healthier lives - the *compression of morbidity scenario*, longer but more disabled/ ill-health lives - the *expansion of morbidity scenario*, or something in between - the *dynamic equilibrium scenario*[5,6]. In order to examine these different hypotheses, life expectancy per se is not a sufficient indicator and needs to be completed with a level of health status. Lifespan without or with disability/ill-health defines the average number of years a person at a certain age is expected to live in the particular health condition. Health expectancies, combining life expectancy with a concept of health - chronic disease, functional limitations, activity restrictions, physical, mental or social well-being - have become essential indicators of the health of the ageing populations, where the quality of remaining life is considered to be equally important as the quantity [7,8]. There are as many possible health expectancies as relevant health indicators [9]. Disability-free life expectancies (DFLE) are commonly used as to refer to a relevant measure of health of the population, and in particular of older cohorts. For the sake of comparability of data and of the more effective measuring of the health status of all Europeans, the European Commission developed a Healthy Life Years (HLY) indicator which is the part of the family of DFLE, being based on a general activity limitation indicator (GALI) [10], and introducing a concept of quality of life [9]. The HLY was presented in the set of structural indicators selected and defined to help measure progress of the 2000 Lisbon strategy objectives [11].

Realising the importance of health as a determinant and a driver of economic growth and competitiveness, the European Commission decided to include public health policy into its economic Lisbon Agenda [12]. HLY indicator was introduced to monitor health as an economic/productivity and societal welfare factor [12].

The Europe 2020 strategy, a successor of the Lisbon Strategy, therefore, highlights the ageing of the EU population as one of pressing societal challenges, calling for actions to foster active and healthy ageing. Health and healthy population is fundamental to the pursuit of smart, sustainable and inclusive growth and better jobs [13].

In one of its flagship initiatives – Innovation Union - Europe 2020 proposed launching a European Innovation Partnership on Active and Healthy Ageing that aims to address the challenge of ageing through innovation [14]. The Partnership sets a headline target to increase HLY at birth on EU average by 2 years by 2020. It is an ambitious yet firm health goal that strives to reduce the socio-economic risks associated with demographic change and to underpin quality of life of all Europeans and especially the older Europeans.

The objective of this paper is to provide analytical research that supported the European Commission in setting the target of increasing healthy lifespan of Europeans by 2 years by 2020. Similarly to life expectancy projections predicting an increasing trend for the next few decades, the paper explores the possible effect of continually postponing death on the overall prevalence of morbidity and disability. It explores three scenarios of HLY trends - compression of morbidity, expansion of morbidity, intermediary dynamic equilibrium - which give a range of possible values to be achieved by 2020 on the basis of which the Partnership selected the goal to be pursued.

The first scenario, proposed by Fries [15,16] assumes that life expectancy is reaching its limit, and the period of ill-health and disability before death is shortened. This theory has two parts: delays in the onset of chronic disease/disability in later life, and one stage in the progression of chronic disease [17]. Accordingly, morbidity and disability are gradually compressed into the shorter span between the increasing age at onset of morbidity and the age at death, and the number of years spent with diseases or disability decreases over time.

The expansion of morbidity hypothesis, developed by Gruenberg and Kramer [18,19] states that mortality reductions will produce more years with morbidity and related disability. The decline in mortality is largely due to the decreasing fatality rate of diseases, rather than a reduction in their incidence. The final stage of the progress of fatal chronic disease is delayed and mainly due to life-sustaining medical interventions. Consequently, declining mortality from fatal diseases does expand longevity but with a substantial increase in the population at high risk of chronic morbidity and related disability. This induces a shift in the distribution of causes of disability from fatal toward less fatal or nonfatal diseases.

This alternative intermediate hypothesis, suggested by Manton [20], states that there exists equilibrium between life expectancy and the health and functioning of the elderly population. In this scenario increased survival does produce an increase in years with morbidity, but years with severe morbidity and disability are relatively constant, because the pace of progression of chronic diseases and disability is reduced. In other words, the proportion of a life span lived with serious illness or disability decreases, whereas the proportion with moderate disability or less severe illness increases. As declines in the rate of disease progression delay the onset of more serious disease states, the dynamic equilibrium scenario implies that mortality reductions will be associated with a redistribution of disease and disability from more to less severe states [5].

The paper does not aim to present complex methodological prediction models. It rather produces a straightforward analysis of HLY projections that helped the European Commission set a firm, politically sound, target. In order to reach that goal, policy makers need to commit to redefining health priorities and goals and developing and implementing relevant strategies and programmes.

## Methods

### Data sources

At the time of drafting the paper, the most recent available data for HLY was from 2008, expect for Belgium, Italy and the UK that provided only data for 2007. 2008 thus is a baseline year for the analysis.

Data on life expectancy (LE) at birth for males and females in 2008 for each Member State were obtained from Eurostat [21]. The projected changes in life expectancy at birth for males and females between 2009 and 2020 were drawn from Eurostat Population Projections 2010-based EUROPOP2010 [22].

Data on healthy life years (HLY) at birth by age, for each Member State and EU27, were drawn from Eurostat [23] that uses the standard Sullivan method for HLY calculation [24]. The prevalence of the health status under consideration in each age group divides the number of person-years into years lived with this status [25]. HLY is based on a Global Activity Limitation Indicator (GALI) question that is a component of the Minimum European Health Module, included in the European Union Statistics of Income and Living Conditions Survey (EU-SILC) [26,27]. The survey is organized by Eurostat. HLY thus becomes a strong indicator allowing for the effective monitoring of levels of health within and between all EU countries in a comparable and consistent way [28]. HLY in comparison to other health expectancy indicators defines healthy condition by the absence of limitations in functioning/disability while explicitly using different levels of health status. Thus it views the health positively [29].

### Methods and calculations

Computations of HLY projections up to 2020 were estimated under three broad scenarios for future health status of the population: (1) the *compression of morbidity*, (2) the *expansion of morbidity,* and (3) the intermediary *dynamic equilibrium.* These drew on theories, as explained above, based on the extent to which health status (or morbidity/disability) of the population may change over time in relation to the growing life expectancy. The scenarios differ in terms of the expected size of the increase in life expectancy and the way in which these mortality reductions might be achieved. For each one a set of assumptions was developed. In all of them, life expectancy was expected to increase according to Eurostat projections. Additionally Scenario 1 assumed that by 2020 HLY would increase by at least the same nominal value as life expectancy and that an increase in life expectancy would be 100% healthy. Scenario 2 assumed that remaining HLY would remain the same for the projected period, and all increase in life expectancy would be 100% with activity limitations. Scenario 3 considered that HLY/LE ratio would remain the same and that not every increase in life expectancy would be healthy.

The analysis is simplified to basic formulas based on data available for LE and HLY. Values for both LE and HLY available on Eurostat – due to data gaps – often limit to individual MSs and rarely provide EU27 average values. Projections of LE referred to individual member states. Therefore EU27 average values of future LE and HLY were computed.

## Results

Life expectancy (LE) at birth has been increasing since decades for both males and females in the EU27. In 2008, the EU27 average LE at birth was estimated to be 74.9 years for males and 81.4 years for females (Table 1). Further gains are projected mostly from lower mortality at older ages. Increasing trends of life expectancy do not however pre-empt a healthy longevity. In 2008 on EU27 average healthy life years (HLY) at birth was estimated as 60.6 years for men and 61.8 years for women (Table 1). The significant gap between HLY and LE exist among all Member States (MSs) for both men and women - in 2008 14.4 years for men and 19.6 for women - as shown in Figures 1 and 2. The healthy years represented around 81% and 76% of the total life expectancy at birth for men and women respectively (Table 1). Values for LE and HLY at birth showed significant differences among Member States, however the spread of HLY at birth was much greater than of LE, observing a gap of nearly 18 years for men and 20 years for women (Table 2).

**Table 1:** Life expectancy and healthy life years at birth within the European Union, 2008 and projections under different scenarios for 2020

**Males**	**2008**				**2020**			**Difference 2008-2020**	
	**LE**	**HLY**	**HLY/LE%**	**LE**	**HLY**	**HLY/LE%**	**LE**	HLY*	HLY/LE%**
Compression	74.9	60.6	80.8	77.0	62.6	81.3	2.1	2.0	0.5
Expansion	74.9	60.6	80.8	77.0	60.6	78.6	2.1	0.0	−2.2
Dynamic Eq	74.9	60.6	80.8	77.0	62.2	80.9	2.1	1.6	0.1
**Females**	**2008**				**2020**			**Difference 2008-2020**	
	**LE**	HLY*	**HLY/LE%**	**LE**	**HLY**	**HLY/LE%**	**LE**	HLY*	HLY/LE%**
Compression	81.4	61.8	75.9	83.0	63.2	76.4	1.6	1.4	0.5
Expansion	81.4	61.8	75.9	83.0	61.8	74.5	1.6	0.0	−1.4
Dynamic Eq	81.4	61.8	75.9	83.0	63.0	75.9	1.6	1.2	0.0

EU average values of LE and HLY = simple mean of values of 27 EU members (2007 values for IT, BE and UK).

*Difference in the HLY: gain/loss of number of years spent healthy.

**Difference in the HLY/LE ratio: gain/loss in the proportion of remaining life spent healthy (HLY/LE%).

**Table 2:** Health inequalities between Member States within the EU measured by HLY gap, 2008 and projections under different scenarios for 2020

**Males**	**2008**			**2020**			**Difference 2008-2020**
	**HLY gap**	**Lowest HLY**	**Highest HLY**	**HLY gap**	**Lowest HLY**	**Highest HLY**	**HLY gap**
Compression	17.7	51.5 LV	69.2 SE	16.3	54.3 LU	70.6 SE	−1.4
Expansion	17.7	51.5 LV	69.2 SE	17.7	51.5 LV	69.2 SE	0.0
Dynamic Eq	17.7	51.5 LV	69.2 SE	16.8	53.6 LU	70.4 SE	−0.9
**Females**	**2008**			**2020**			**Difference 2008-2020**
	**HLY gap**	**Lowest HLY**	**Highest HLY**	**HLY gap**	**Lowest HLY**	**Highest HLY**	**HLY gap**
Compression	19.6	52.3 SK	71.9 MT	18.4	54.3 SK	72.7 MT	−1.2
Expansion	19.6	52.3 SK	71.9 MT	19.6	52.3 SK	71.9 MT	0.0
Dynamic Eq	19.6	52.3 SK	71.9 MT	18.9	53.6 SK	72.5 MT	−0.7

*LV* Latvia, *SE* Sweden, *SK* Slovakia, *MT* Malta, *LU* Luxembourg.

Such inter-country differences in values of LE and HLY across the EU make it difficult to model any EU level projections.

### Compression of morbidity

Under this scenario, the study predicted that life expectancy and HLYs for men would grow on average at nearly same 2-year pace by 2020 (Table 1). For women, LE and HLYs would increase on average by 1.6 and 1.4 years accordingly by 2020. The expected years with disability would remain unchanged (14.4 years for men; 19.8 years for women by 2020). The HLY/LE ratio would improve from 80.8% to 81.3% for men and from 75.9% to 76.4% for women. In relative terms (HLY/LE%), the remaining healthy lifespan would increase only by 0.5% for both men and women. This scenario foresees a very slight reduction of health inequalities among the MSs, namely the HLY gap, which is defined as the difference between the highest and lowest HLY, would decrease by 1.4 years for men and by 1.2 years for women, reaching 16.3 years and 18.4 years respectively (Table 2).

### Expansion of morbidity

Similar to the previous scenario, life expectancy would increase by 2.1 years and 1.6 years for men and women respectively by 2020, while HLYs would remain unchanged (Table 1). The expansion of morbidity would imply an increase in the expected years with disability of 2.1 years in men and 1.6 years in women. This would result in a deterioration of the HLY/LE ratio for both men and women from 80.8% to 78.6% and from 75.9% to 74.5% respectively. The proportion of life in good health would be reduced: 2.2% and 1.4% loss of good health for men and women respectively. This scenario would not change the level of health inequalities among Member States, remaining a gap of 17.7 years for men and 19.6 years for women (Table 2).

### Dynamic equilibrium

Under this scenario, HLY at birth on EU average is expected to increase by 2020 but to a lesser extent as the rise in life expectancy, namely by 1.6 years in men 1.2 years in women (Table 1). This would imply that HLY/LE ratio would remain unchanged for women while would increase by 0.1% for men, and in relative terms this would mean nearly no improvement in healthy life expectancy by 2020. Health inequalities in men would be reduced by 0.9 years, reaching 16.8 years, while in women would increase by 0.7 years, reaching 18.9 years (Table 2).

## Discussion

Increasing trends in longevity and uncertainty in the development of HLY across the EU countries raise the question of whether people will live longer and healthier lives, longer but more disabled lives, or something in between. To challenge this query, the study computed future HLY at birth for the EU27 as whole, based on calculations for individual MSs, under 3 scenarios. This scenario modelling allowed to observe the interplay of changes in mortality and morbidity and disability trends, and to determine whether population health is to improve or deteriorate.

Predictions about the likely effect of the continually delaying death on the period of morbidity and disability at the end of life depend on the causal factors that are driving this trend. As previously emphasised, data limitations make confident interpretation of past trends nearly impossible, thus hindering the robust computing of future scenarios. This means a great level of uncertainty for predicting which scenario might prevail. Difficulties in forecasting the development of health expectancies (here HLY) reinforce the conclusions of other studies which contend that gaps in existing health data impede the modelling on the basis of the past trends of health status and health expectancy [5]. This makes it difficult to establish any coherent set of hypotheses for projections of health status [5]. In addition, other factors that might influence the health of future cohorts (e.g. changes in life style such as higher obesity levels or the opportunity to introduce new medical technologies) were not considered in the study despite the fact that these factors would affect the predicted HLY under the different scenarios.

### Compression of morbidity

The results of this scenario illustrated the future potential for health improvement from policies that increase healthy life years. Areas of intervention, that are covered by the Partnership, should include preventive strategies for healthy or healthier lifestyles and preventive measures to combat chronic diseases postponing the onset of age-associated diseases, and allowing for an entire plausibility of the compression of morbidity scenario.

### Expansion of morbidity

This scenario’s outcomes presented the potential of medical and care advances in reducing fatality rates for chronic diseases while holding disease incidence and underlying patterns of the disease constant. This results in a longer survival with advanced degenerative and disabling diseases so that the period of time that people spend in a state of chronic ill-health and disability at the end of the life increases. An increase in life expectancy, in this case mainly driven by the growing and innovative capabilities of medicine and care to prevent fatal outcomes from degenerative diseases, creates pressure on health and social care services. It is also a burden on carers and communities, as greater numbers suffer chronic disease and disability.

### Dynamic equilibrium

The outcomes envisaged under this hypothesis in terms of an increase in life expectancy as well as better health would be possible if medical interventions and advances as well as lifestyle changes were put in place in an earlier (less severe) stage of the disease process. Consequently, due to improved secondary prevention, among others, long term social care costs would not have to experience greater pressure.

**Figure 3:**
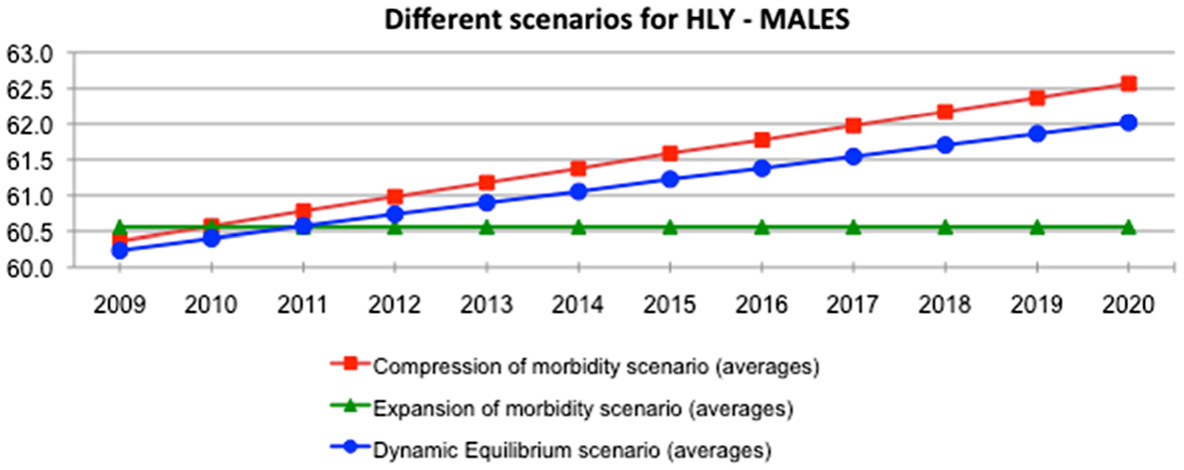
Scenarios for the future male population health by Healthy Life Years (HLY) in the European Union, 2009-2020.

**Figure 4:**
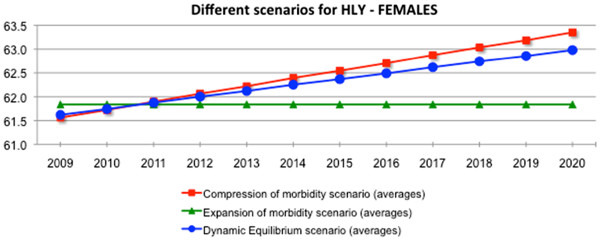
Scenarios for the future female population health by Healthy Life Years (HLY) in the European Union, 2009-2020.

Figures 3 and 4 summarise the analysis of the 3 scenarios, illustrating the direction of expected change of healthy life years over the next 10 years. The plausibility in achieving the target of improved lives spent in good health under 2 scenarios - the compression of morbidity and the dynamic equilibrium - can be clearly depicted. These two scenarios predict an increase of HLY at EU average level by 1.6 to 2 years for men and 1.2 to 1.4 years for women This is however, under the condition that relevant policy intervention and action, including health promotion and preventive action and the use of medical and care advances, is implemented. Also, heterogeneity of developments of HLY among individual countries of the EU calls for intra-country analysis and setting of relevant policy measures.

The results of the study triggered the political decision of setting the global target of 2 additional HLY for the European Innovation Partnership on Active and Healthy Ageing to be achieved within a decade.

## Conclusions

The analysis needs to be considered with a high level of prudence and bearing in mind a margin of error. A series of other factors, as mentioned above, exist that might impact people’s life expectancies, mortality and morbidity rates and have not been considered.

Following results observed under the different scenarios (Table 1), the potential HLY target could foresee an increase by 2 years for men and 1.4 years for women at the EU level. It is a ‘grand’ goal but can be reached in next couple of years.

However, the big challenge is how to reflect MS levels of health development in the EU average health indicator. Statistics clearly show that EU countries characterise very different levels of health progress, with a gap of 2 decades and diverging trends. With this in mind, the EU HLY target should be complemented by national HLY targets for men and women, set by MSs. It would make MSs feel equally responsible for the delivery of the HLY target, regardless of their starting positions. In addition, accompanying the EU headline target with national targets would be in line with the Europe 2020 approach breaking with ‘one size fits all’ approach.

There is an urgent need for action and intervention at different levels in order to close a gap between a number of life years and those lived in good health, disability or frailty free.

It should be nevertheless emphasized that the identification of HLY as a headline target for one of Europe 2020 key initiatives is a move forward towards development of comparable, robust and sustainable health indicators. Given the multifaceted goals of the Partnership aiming to improve not only health status but also quality of life, this initiative is a great opportunity to develop a comprehensive monitoring framework based on a set of indicators that monitor health, quality of life, while supporting active ageing and employment in the context of lengthening of life, with sound and comparable, less subjective data.

In conclusion, the HLY indicator offers the means to monitor whether and to what extent the reduction of the longevity gaps in the EU and the increase in life expectancy impact better functional health and better quality of life. HLY developments can also support in setting up adequate policy measures helping to compress health expectancy gaps across EU countries and between genders [3,30].

## Competing interests

The author declares not to have any financial or personal relationship with other people or organisations that could bias her work.

## Authors’ contributions

The author carried out the data analysis, conceived the study and drafted the manuscript with the analysis and interpretation of the results.
